# Costs and healthcare utilisation of patients with heart failure in Spain

**DOI:** 10.1186/s12913-020-05828-9

**Published:** 2020-10-20

**Authors:** Carlos Escobar, Luis Varela, Beatriz Palacios, Margarita Capel, Antoni Sicras, Aram Sicras, Antonio Hormigo, Roberto Alcázar, Nicolás Manito, Manuel Botana

**Affiliations:** 1 University Hospital La Paz, Madrid, Spain; 2 AstraZeneca Spain, Barcelona, Spain; 3 Health Economics and Outcomes Research, Atrys Health, Barcelona, Spain; 4 Primary Care Center Salud Puerta Blanca, Malaga, Spain; 5 University Hospital Infanta Leonor, Madrid, Spain; 6 Hospital de Bellvitge, Hospitalet de Llobregat, Barcelona, Spain; 7 Hospital Universitario Lucus Augusti, Lugo, Spain

**Keywords:** Heart failure cost, Dapagliflozin, DAPA-HF, Heart failure, Hospitalization, Sacubitril/valsartan

## Abstract

**Background:**

Increasing the knowledge about heart failure (HF) costs and their determinants is important to ascertain how HF management can be optimized, leading to a significant decrease of HF costs. This study evaluated the cumulative costs and healthcare utilisation in HF patients in Spain.

**Methods:**

Observational, retrospective, population-based study using BIG-PAC database, which included data from specialized and primary care of people ≥18 years, from seven autonomous communities in Spain, who received care for HF between 2015 and 2019. The healthcare and medication costs were summarized on a yearly basis starting from the index date (1st January 2015), and then cumulatively until 2019.

**Results:**

We identified 17,163 patients with HF (year 2015: mean age 77.3 ± 11.8 years, 53.5% men, 51.7% systolic HF, 43.6% on NYHA functional class II). During the 2015–2019 period, total HF associated costs reached 15,373 Euros per person, being cardiovascular disease hospitalizations the most important determinant (75.8%), particularly HF hospitalizations (51.0%). Total medication cost accounted for 7.0% of the total cost. During this period, there was a progressive decrease of cardiovascular disease hospital costs per year (from 2834 Euros in 2015 to 2146 Euros in 2019, *P* < 0.001), as well as cardiovascular and diabetic medication costs.

**Conclusions:**

During the 2015–2019 period, costs of HF patients in Spain were substantial, being HF hospitalizations the most important determinant. Medication costs represented only a small proportion of total costs. Improving HF management, particularly through the use of drugs that reduce HF hospitalization may be helpful to reduce HF burden.

**Supplementary information:**

**Supplementary information** accompanies this paper at 10.1186/s12913-020-05828-9.

## Background

Heart Failure (HF) is a complex clinical syndrome caused by structural or functional cardiac alterations, leading to a reduced cardiac output or elevated intracardiac pressures at rest or during stress, that cause the typical symptoms such as breathlessness, or fatigue [1, 2]. HF is a growing problem worldwide [3]. It has been estimated that the prevalence of HF is around 2% of the adult population in developed countries, raising to more than 10% in those individuals aged 70 years or older [4]. In Spain, it has been reported a higher prevalence of HF, reaching 5% in some studies [5, 6]. Of note, it is expected that the prevalence of HF will increase in the following years, mainly due to the ageing of the population, the rise in HF risk factors (i.e. hypertension, diabetes) and the better treatment of acute cardiovascular events [3].

Despite traditional HF therapies (i.e renin angiotensin system inhibitors, beta blockers and aldosterone antagonists), mortality and hospitalization rates remain unacceptably high [7, 8]. Thus, the MAGGIC meta-analysis that included individual data on 39,372 patients with HF, from 30 cohort studies showed that 40.2% of patients died after 2.5 years of follow-up [8]. However, in the last years, the PARADIGM-HF and more recently, the DAPA-HF trials have shown that sacubitril/valsartan and dapagliflozin, respectively, have a positive impact on morbidity and mortality among patients with HF and reduced left ventricular ejection fraction [9, 10].

Remarkably, HF represents a major and growing economic problem [3, 11, 12]. Studies particularly focused on HF economic burden are important as they contribute to a better understanding of the drivers and problems which may lead to the increasing HF costs [3]. Increasing the knowledge about HF costs and their determinants is important in order to ascertain how HF management can be optimized, leading to a significant decrease of HF costs [3, 7, 11, 12]. Unfortunately, data about costs of HF in Spain are very scarce or limited to the use of specific drugs, but not focused on a comprehensive approach [13–18]. For example, a study performed in Spain in 2014 among only 374 patients reported a high total cost associated with HF [18]. As a result, new studies with a high number of patients that provide current data are warranted.

The aim of this study was to evaluate the cumulative costs and healthcare utilisation in HF patients in Spain over the last 5 years, along with the epidemiological characterization of the population at index date (1st January 2015). This was also analyzed in a population who met the most relevant inclusion criteria of the DAPA-HF trial [10] (DAPA-HF like population) with the aim of understanding the costs associated with the management of HF with reduced ejection fraction from the DAPA-HF trial population.

## Methods

Observational cohort study, comprising cross-sectional and longitudinal retrospective analyses using secondary data captured in electronic health records from seven Spanish regions. Data sources were provided by BIG-PAC®. BIG-PAC is an electronic database that integrate information from primary and specialist care medical records. This database has been validated as an information source for studies of epidemiology, therapeutic adaptation and health/non-healthcare resource use and associated costs. It is representative of the Spanish population [19].

The HF population was defined as all patients ≥18 years of age and with at least one diagnosis of HF prior to the index date (first January 2015). Type 2 diabetes (T2D) was defined as all patients ≥18 years of age filling a prescription of any antidiabetic medication, T2D diagnosis or HbA1c > 7% prior to index date. The DAPA-HF like population included those patients ≥18 years, with a HF history of more than 8 weeks, treatment with device therapy or standard HF treatment, NYHA class ≥II within 1 year prior to index date and left ventricular ejection fraction ≤40%. Patients with HF hospitalization in the previous 4 weeks prior to index date, myocardial infarction, unstable angina pectoris, stroke, transient ischemic attack, coronary revascularization or implantation of therapeutic device < 12 weeks prior to index date, were excluded from the population.

Comorbidities were searched for in all available data prior to index date, excepting for severe hypoglycemia that was considered only within 1 year before index date and cancer which was searched for up to 5-year prior to index date. A minimum of 1 year of data before index date was required. Comorbidities included cardiovascular disease (myocardial infarction, percutaneous or surgical revascularization, unstable angina, angina pectoris), HF, chronic kidney disease, atrial fibrillation, stroke (hemorrhagic, ischemic, transitory ischemic attack), peripheral artery disease, major organ specific bleeding, microvascular complications (diabetic mono−/polyneuropathy, diabetic eye complications, diabetic kidney disease), severe hypoglycemia, cancer, chronic obstructive pulmonary disease, and lower limb complications. ICD-9 and ICD-10 codes (https://eciemaps.mscbs.gob.es) were considered for the diagnosis of comorbidities (supplementary Table 1).

The information about treatment was recorded from the registries for dispensing medicines, according to the Anatomical Therapeutic Chemical Classification System (supplementary Table 1) [20]. HF treatments (angiotensin-converting enzyme inhibitors, angiotensin receptor blockers, beta blockers, aldosterone antagonists, sacubitril/valsartan, loop diuretics, digoxin), warfarin, statins, aspirin, receptor P2Y12 antagonists, calcium channel blockers, thiazides, nitrates, antidiabetic medications (SGLT-2 inhibitors, metformin, sulfonylurea, DPP-4 inhibitors, GLP-1 receptor agonists, metiglinides, glitazones, acarbose, miglitol, insulin) were recorded. The prescription of a drug in a specific patient was based only on medical criteria (clinical practice).

Prevalence, incidence and baseline characteristics (total HF population and by T2D status), including demographics, comorbidities and medications were calculated at index date (first January 2015).

The healthcare resource use and costs and medication costs were summarized for the total HF population on a yearly basis starting from index date (first January 2015), and then cumulatively until the end of the last year of follow up (31st December 2019). All hospital visits (total and cardiovascular events), the number of medical visits and emergency room visits and medication costs (total, cardiovascular related, HF related and diabetes related) were included for the analysis of the annual direct healthcare costs. Patients who died during follow-up had a cost of 0 allocated to the remaining duration of the study, whereas a patient leaving the database prior to data cut off was not included in the denominator for the time after leaving the database. Annual indirect non-health costs included the number of days of productivity lost due to disability.

Rates were obtained from hospital accounting, except for the medication and indirect costs which were calculated as follows, respectively: a) medical prescriptions: according to the retail price per package at the time of dispensing [21]; b) costs for days of productivity lost: according to the mean interprofessional wage [22]. Hospital admission costs for cardiovascular events during follow-up were obtained taking into consideration daily hospital rate and number of hospital days per stay. Rates are summarized in supplementary Table 2.

### Statistical analysis

Categorical variables were described by their absolute (n) and relative frequencies (%). Continuous variables were described using the mean and standard deviation. Categorical variables were compared with the Chi-square test or the Fisher exact test when appropriate. When two means were compared, the t-student test was used. Analyses of health care cost were performed for the index date with 5 year of follow-up. The cumulative mean healthcare cost was estimated and presented on a yearly basis from the index date until last year of follow-up. Health care costs were presented per patient (mean cost). A level of statistical significance of 0.05 was applied in all the statistical tests. The data were analyzed using the statistical package SPSS v22.0 (SPSS Inc., Chicago, Illinois, USA).

## Results

**Fig. 1:**
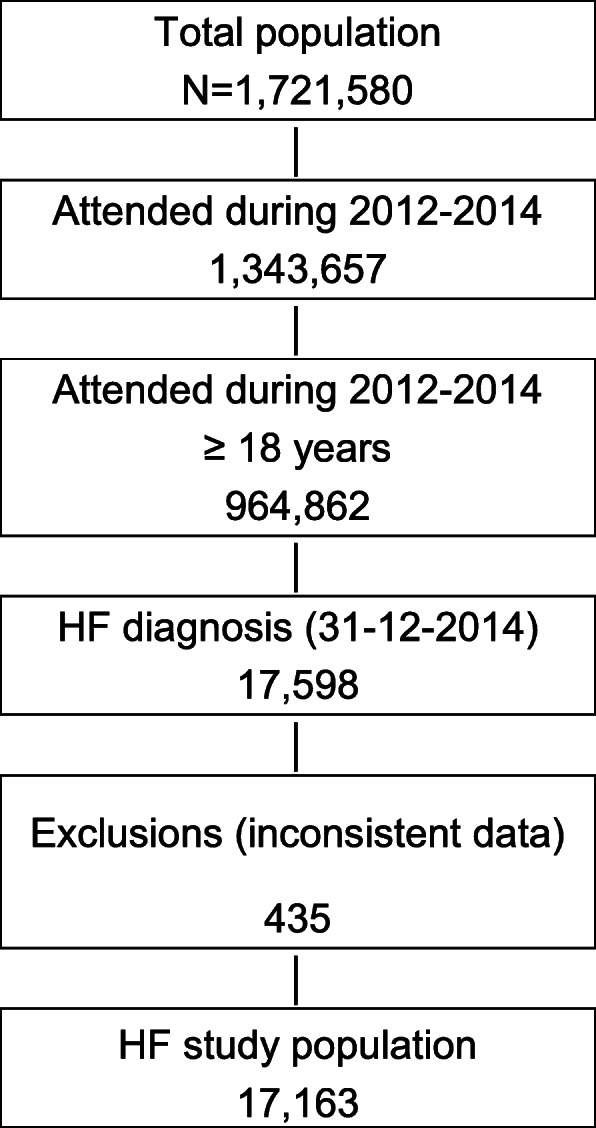
Flowchart cost population (2015)

Out of 1,7 millions of persons included in the BIG-PAC® database in 2015, 1,3 million people were attended during the 2012–2014 period, of whom 964,862 were 18 years or older. At index date, 17,598 patients had HF. As 435 patients were excluded due to inconsistent data, 17,163 patients (1.78%) comprised the HF study population (Fig. 1). Incidence at index date was 2.84 × 1000 patient-years.

The baseline clinical characteristics of the HF population according to the presence of T2D were presented in Table 1. Overall, mean age was 77.3 ± 11.8 years, 53.5% of patients were men, 51.7% of patients had reduced left ventricular ejection fraction HF, and the majority of patients were on NYHA functional class II (43.6%) or III (36.1%). A total of 5815 (33.9%) patients had T2D. The presence of other comorbidities was common: 30.0% of patients had atrial fibrillation, 29.5% chronic kidney disease, 23.3% ischemic heart disease, 17.2% chronic obstructive pulmonary disease, 12.8% cancer and 9.5% previous stroke. Two thirds of patients were taking renin angiotensin system inhibitors, 68.3% beta blockers, 30.0% aldosterone antagonists, and 8.5% sacubitril/valsartan. The baseline clinical profile of patients according to the presence of T2D was also compared. The percentage of patients with left ventricular ejection fraction ≤40% was higher in patients with type 2 diabetes (53.1% vs 51.0%; *P* = 0.015). Those patients with T2Dd had more ischemic heart disease, stroke, atrial fibrillation, peripheral artery disease, and chronic kidney disease, and a higher body mass index (*P* < 0.05 or less for all comorbidities).

**Table 1:** Baseline clinical characteristics of the heart failure population at index date 1st January 2015 according to the presence of type 2 diabetes

	No T2D (***n*** = 11,348; 66.1%)	T2D (***n*** = 5815; 33.9%)	Total HF (***n*** = 17,163; 100%)	P*
**Biodemographic data**				
Age, years	77.4 ± 12.6	77.2 ± 10.1	77.3 ± 11.8	0.236
≥ 85 years, n (%)	6308 (55.6)	2648 (45.5)	8956 (52.2)	< 0.001
Gender, male, n (%)	6052 (53.3)	3135 (53.9)	9187 (53.5)	0,470
Body mass index, Kg/m^2^	28.1 (5.8)	29.5 (6.0)	28.6 (5.9)	< 0.001
Systolic blood pressure (mmHg)	128.7 ± 21.9	131.8 ± 22.6	129.8 ± 22.2	< 0,001
**Heart failure data**				
NYHA functional class, n (%)				
I	1321 (11,6)	654 (11.3)	1975 (11.5)	
II	5008 (44.1)	2476 (42.6)	7484 (43.6)	0.001
III	4068 (35.9)	2135 (36.7)	6203 (36.1)	
IV	395 (3.5)	268 (4.6)	663 (3.9)	
Left ventricular ejection fraction, %	44.2 ± 10.6	41.5 ± 12.4	43.3 ± 11.3	< 0.001
≤ 40%, n (%)	5217 (51.0)	2798 (53.1)	8015 (51.7)	0.012
> 40 - < 50%, n (%)	801 (7.8)	428 (8.1)	1229 (7.9)	0.012
≥ 50%, n (%)	4217 (41.2)	2039 (38.7)	6256 (40.4)	0.012
**Laboratory data**				
eGFR, ml/min/1.73 m^2^	76.1 ± 20.4	72.3 ± 20.4	74.8 ± 20.5	< 0.001
HbA1c, %	5.2 ± 0.9	7.3 ± 0.8	6.1 ± 1.4	< 0.001
**Comorbidities**				
**Cardiovascular disease, n (%)**				
Ischemic heart disease	2174 (19.1)	1834 (31.5)	4006 (23.3)	< 0.001
Myocardial infarction	1350 (11.9)	1126 (19.4)	2466 (14.4)	< 0.001
CABG	122 (1.1)	132 (2.3)	254 (1.5)	0.001
PCI with stent	285 (2.5)	243 (4.2)	528 (3.1)	< 0.001
Unstable angina	414 (3.7)	384 (6.6)	798 (4.7)	< 0.001
Angina pectoris	686 (6.1)	493 (8.5)	1179 (6.9)	< 0.001
Stroke	1015 (8.9)	622 (10.7)	1637 (9.5)	< 0.001
Ischemic stroke	779 (6.9)	467 (8.0)	1246 (7.3)	0.005
Hemorrhagic stroke	72 (0.6)	28 (0.5)	100 (0.6)	0.213
Transitory ischemic attack	256 (2.3)	173 (3.0)	429 (2.5)	0.004
Atrial Fibrillation	3339 (29.4)	1805 (31.0)	5144 (30.0)	0.029
Peripheral artery disease	500 (4.4)	326 (5.6)	826 (4.8)	0.001
Chronic kidney disease	2819 (24.8)	2236 (38.5)	5055 (29.5)	< 0.001
Microvascular complications	0	1810 (31.1)	1810 (10.5)	< 0.001
Diabetic mono−/polyneuropathy	0	380 (6.5)	380 (2.2)	< 0.001
Diabetic eye complications	0	1598 (27.5)	1849 (10.8)	< 0.001
Diabetic foot/peripheral angiopathy	0	251 (4.3)	264 (1.5)	< 0.001
Diabetic kidney disease	0	468 (8.1)	521 (3.0)	< 0.001
Diabetes with complications	0	3207 (55.2)	3452 (20.1)	< 0.001
Severe hypoglycemia	0	461 (7.9)	461 (2.7)	< 0.001
**Other comorbidities, n (%)**				
Cancer	1465 (12.9)	733 (12.6)	2198 (12.8)	0.572
Chronic obstructive pulmonary disease	1928 (17.0)	1027 (17.7)	2955 (17.2)	0.270
Lower limb amputations	37 (0.3)	108 (1.9)	145 (0.8)	< 0.001
Major organ specific bleeding	111 (1.0)	177 (3.0)	288 (1.7)	< 0.001
**Therapies**				
**Heart failure medications, n (%)**	11,348 (100)	5815 (100)	17,163 (100)	**–**
Renin angiotensin system inhibitors	6973 (61.5)	4452 (76.6)	11,425 (66.6)	< 0.001
Angiotensin-converting enzyme inhibitors	3354 (29.6)	1986 (34.2)	5340 (31.1)	< 0.001
Angiotensin receptor blockers	3619 (31.9)	2466 (42.4)	6085 (35.5)	< 0.001
Beta blockers	7509 (66.2)	4218 (72.5)	11,727 (68.3)	< 0.001
Loop-diuretics	7416 (65.4)	4645 (79.9)	12,061 (70.3)	< 0.001
Aldosterone antagonists	3424 (30.2)	1728 (29.7)	5152 (30.0)	0.537
Sacubitril/valsartan	987 (8.7)	477 (8.2)	1464 (8.5)	0.272
Digoxin	799 (7.0)	398 (6.8)	1197 (7.0)	0.632
**Other cardiovascular medications, n (%)**				
Low dose aspirin	3017 (26.6)	2037 (35.0)	5054 (29.5)	< 0.001
Receptor P2Y12 antagonists	1177 (10.4)	634 (10.9)	1811 (10.6)	0.284
Statins	5928 (52.2)	3238 (55.7)	9166 (53.4)	< 0.001
Antihypertensives	2415 (21.3)	1478 (25.4)	3893 (22.7)	< 0.001
Dihydropyridines CCB	1564 (13.8)	1027 (17.7)	2591 (15.1)	< 0.001
Thiazides	520 (4,6)	268 (4,6)	788 (4.6)	0,938
Non-hydropyridines CCB	504 (4,4)	290 (5,0)	794 (4.6)	0,107
Nitrates	1169 (10,3)	788 (13,6)	1957 (11,4)	< 0,001
Warfarin	2438 (21,5)	1464 (25,2)	3902 (22,7)	< 0,001
**Diabetes medications, n (%)**	0	5383 (92.6)	5383 (31.4)	< 0.001
Metformin	0	3862 (66.4)	3862 (22.5)	< 0.001
Sulfonylurea	0	1952 (33.6)	1952 (11.4)	< 0.001
DPP4 inhibitors	0	1416 (24.4)	1416 (8.3)	< 0.001
SGLT-2 inhibitors	1 (0%)	2 (0)	3 (0)	< 0.001
GLP-1 receptor agonists	0	57 (1.0)	57 (0.3)	< 0.001
Metiglinides	0	319 (5.5)	319 (1.9)	< 0.001
Glitazones	0	57 (1.0)	57 (0.3)	< 0.001
Acarbose	0	88 (1.5)	88 (0.5)	< 0.001
Insulin	0	1367 (23.5)	1367 (8.0)	< 0.001

*T2D* Type 2 diabetes, *HF* Heart failure, *eGFR* Estimated glomerular filtration rate, *CABG* Coronary artery bypass graft, *PCI* Percutaneous coronary intervention, *CCB* Calcium channel blockers, *DPP4* Dipeptidyl peptidase 4, *SGLT-2* Sodium-glucose Cotransporter-2, *GLP-1* Glucagon-like peptide-1

*p values comparing no T2D vs T2D

**Fig. 2:**
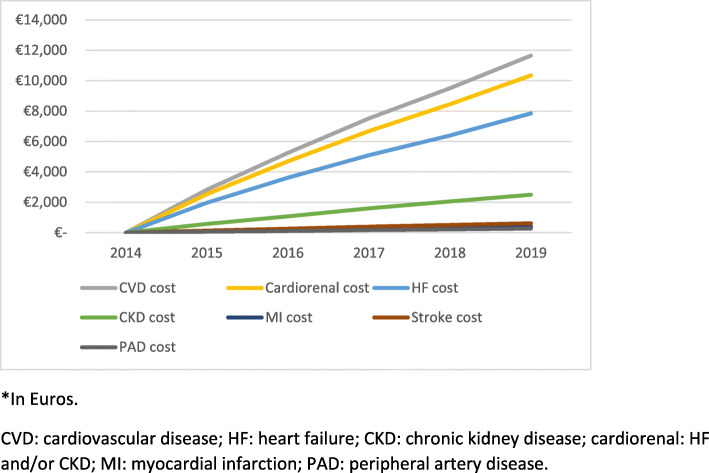
Patient cumulative hospital mean cost*. *In Euros. CVD: cardiovascular disease; HF: heart failure; CKD: chronic kidney disease; cardiorenal: HF and/or CKD; MI: myocardial infarction; PAD: peripheral artery disease

**Fig. 3:**
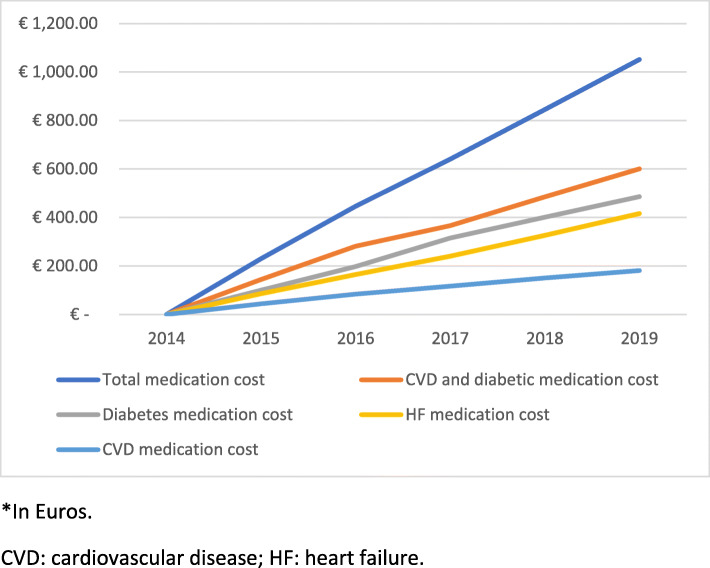
Patient cumulative medication mean cost*. *In Euros. CVD: cardiovascular disease; HF: heart failure

Patient hospital mean cost for year was presented in Table 2. In general, from 2015 to 2019, there was a progressive decrease of cardiovascular disease hospital cost per patient year (from 2834 to 2146 Euros, *P* < 0.001). Overall, patient cumulative cardiovascular disease hospital mean cost reached 11,649 Euros in 2019 (supplementary Table 3 and Fig. 2). The great burden for this cost was due to cardiorenal (HF and/or chronic kidney disease) hospitalizations (88.8% of the total cost), particularly HF (67.3% of the total cost). With regard to medication, from 2015 to 2019, diabetes medication mean cost varied from 101 to 85 Euros (*P* < 0.001) per patient and year and HF medication mean cost from 86 to 90 Euros (P < 0.001), respectively (Table 2). The cumulative mean cost of diabetes medication and HF medication reached 486 and 417 Euros, respectively, in 2019 (supplementary Table 3 and Fig. 3).

**Table 2:** Patients hospital mean cost for year^a^

	2015		2016		2017		2018		2019		Cumulative cost in 2019
	mean	SD	mean	SD	mean	SD	mean	SD	mean	SD	
**Total hospital cost**											
CVD	2834	5212	2416	5065	2268	4839	1985	4937	2146	4947	11,649
Cardiorenal	2536	4859	2155	4565	2001	4433	1759	4329	1896	4460	10,346
HF	1967	4094	1657	3814	1476	3609	1297	3505	1447	3604	7842
CKD	569	2173	498	2024	525	2057	463	1943	450	1968	2504
MI	98	812	87	800	79	760	70	776	83	761	416
Stroke	138	970	126	942	132	977	108	940	113	887	616
PAD	63	740	49	619	57	682	48	660	54	602	271
**Medication cost**											
Total medication	230	425	216	426	227	426	204	432	206	421	1083
Diabetes medication	101	336	97	336	118	324	85	308	85	317	486
HF medication	86	130	79	131	76	203	86	268	90	313	417
CVD medication	44	96	40	91	33	80	33	86	31	339	181

*CVD* Cardiovascular disease, *HF* Heart failure, *CKD* Chronic kidney disease, *cardiorenal* HF and/or CKD, *MI* Myocardial infarction, *PAD* Peripheral artery disease

^a^In Euros

The health resources use for each year, including primary care visits, specialized visits, emergency rooms visits, hospitalization and diagnostic tests, was shown in Table 3. The proportion of hospitalized patients decreased from 31.5% in 2015 to 22.9% in 2019 (*P* < 0.001), the days for hospitalized patients due to HF from 8.3 to 7.2 days (P < 0.001), and the proportion of patients that died from 9.4 to 4.9% (P < 0.001), respectively. Total sanitary cost decreased from 3700 Euros in 2015 to 2770 Euros in 2019 (P < 0.001). Including indirect costs, total cumulative patient mean costs reached 15,373 Euros in 2019, 263,852,978 Euros per total HF population (Table 4).

**Table 3:** Health resources use for each year per patient

	2015		2016		2017		2018		2019	
	mean	SD	mean	SD	mean	SD	mean	SD	mean	SD
Primary care visits, mean (SD)	12.7	15.0	10.9	14.2	9.6	13.9	8.8	14.3	7.7	13.0
Laboratory requests, mean (SD)	0.6	1.2	0.6	1.2	0.6	1.2	0.7	1.6	1.0	2.1
Radiology and other tests, mean (SD)	0.6	1.2	0.6	1.2	0.6	1.2	0.7	1.6	1.0	2.2
Specialized visits, mean (SD)	2.1	4.2	1.3	4.9	1.1	4.8	1.2	4.9	1.1	4.8
Emergency rooms visits, mean (SD)	0.8	2.5	0.7	3.3	0.7	3.1	0.5	2.3	0.5	3.3
Hospitalization										
Days, mean (SD)	5.9	10.8	5.0	10.5	4.7	10.0	4.1	10.3	4.5	10.3
Hospitalized patients, n (%)	5399 (31.5)		4557 (26.6)		4227 (24.6)		3628 (21.1)		3923 (22.9)	
Days for hospitalized patients due to heart failure only, mean (SD)	8.3	10.7	7.7	10.6	7.5	10.1	6.9	10.1	7.2	10.2
Frequency of hospitalization, n (%)										
0	11,764 (68.5)		12,606 (73.5)		12,936 (75.4)		13,535 (78.9)		13,240 (77.1)	
1	4243 (24.7)		3565 (20.8)		3225 (18.8)		2780 (16.2)		2914 (17.0)	
2	1001 (5.8)		855 (5.0)		867 (5.1)		724 (4.2)		850 (5.0)	
3+	156 (0.9)		137 (0.8)		137 (0.8)		124 (0.7)		160 (0.9)	
Disability										
Days of disability, mean (SD)	0.4	5.9	0.4	6.0	0.6	10.9	0.4	5.7	0.4	5.1
Patients with disability, n (%)	158 (0.9)		132 (0.8)		134 (0.8)		140 (0.8)		190 (1.1)	
Average days of sick leave (disability only), mean (SD)	43.8	43.5	47.4	49.5	77.2	96.9	50.5	38.5	36.7	32.6
Mortality, n (%)	1608 (9.4)		1259 (7.3)		927 (5.4)		893 (5.2)		839 (4.9)	

All years calculated with 17.163 patients

**Table 4:** Patients total mean cost for year and cumulative cost in 2019^a^

	2015		2016		2017		2018		2019		Cumulative cost in 2019
	mean	SD	mean	SD	mean	SD	mean	SD	mean	SD	
Cost of primary care visits	308	362	264	344	231	336	214	345	186	315	1202
Cost of laboratory requests	20	39	19	38	18	37	24	50	32	69	113
Cost of radiology and other tests	24	46	22	45	21	45	28	59	37	80	131
Cost of specialized visits	195	392	123	467	108	451	108	462	101	452	635
Cost of emergency rooms visits	89	291	81	394	79	363	57	276	61	382	368
Cost of Hospitalization	2834	5212	2416	5065	2268	4839	1985	4937	2146	4947	11,649
Cost of medication	230	425	216	426	227	426	204	432	206	421	1083
Sanitary Cost	3700	5623	3141	5516	2920	5347	2620	5153	2770	5181	15,151
Indirect Cost/Sick Leave	41	597	37	606	61	1.104	42	578	41	520	222
**Total Cost**	**3741**	**5665**	**3178**	**5553**	**2981**	**5482**	**2662**	**5202**	**2811**	**1276**	**15,373**

^a^In Euros

A specific analysis was performed in the DAPA-HF like population (*n* = 3178). In this subpopulation, mean age was 76.9 ± 11.7 years, 51.4% were men, and all patients had reduced left ventricular ejection fraction HF (mean left ventricular ejection fraction 34.5 ± 7.9%). The majority of patients were on NYHA functional class II (52.1%) or III (43.0%). A total of 1314 (41.3%) patients had T2D. With regard HF medication, 77.0% of patients were taking renin angiotensin system inhibitors, 100% beta blockers, 21.0% aldosterone antagonists, and 8.8% sacubitril/valsartan. Compared with patients without diabetes, those patients with T2D were taking more renin angiotensin system inhibitors, aldosterone antagonists and sacubitril/valsartan (supplementary Table 4).

With regard to patients hospital mean cost for year for this subpopulation, there was a progressive decrease of cardiovascular disease hospital cost per year (from 3269.6 Euros in 2015 to 2539.5 Euros in 2019, *P* < 0.001). Overall, patient cumulative cardiovascular disease hospital mean cost reached 13,775 Euros in 2019. The great burden for this cost was due to cardiorenal hospitalizations (87.7% of the total hospital cost), particularly HF (65.9% of the total hospital cost). With regard to medication, from 2015 to 2019, diabetes medication mean cost decreased from 128.5 to 74.6 Euros (P < 0.001) and HF medication mean cost from 112.9 to 74.7 Euros (P < 0.001), respectively. The cumulative mean cost of diabetes medication and HF medication reached 540 and 514 Euros, respectively, in 2019 (supplementary Table 5).

## Discussion

Our study showed that in Spain, during the 2015–2019 period HF associated costs were high (patient total cost of 15,373 Euros), being cardiovascular hospitalizations the most important determinant (75.8%), particularly HF hospitalizations (51.0%). Total medication cost accounted for 7% of the total HF cost. In addition, the annual cardiovascular hospitalization mean cost progressive decreased over time.

In our study, the prevalence of HF was about 1.8%. With regards to the HF population, mean age was 77 years, around half of patients had systolic HF, the majority of patients were on NYHA functional class II or III, one third had diabetes and comorbidities were common. In Spain, the studies performed in different clinical settings (hospital and outpatients) show a higher prevalence of HF [23]. However, the population-based studies reported similar numbers to our study [24]. The proportion of patients with systolic HF, as well as the presence of numerous comorbidities are in line with previous studies [5, 24]. As a result, our study can be considered fairly representative of the Spanish population.

With regards to HF therapies, approximately two thirds of patients were taking renin angiotensin system inhibitors and beta blockers, nearly one third aldosterone antagonists, and only 8.5% sacubitril/valsartan. The optimization of treatment of patients with HF is necessary not only to improve functional class and quality of life, but also to reduce morbidity and mortality [1, 2]. These numbers are lower than those reported in HF units, but in line with those from other clinical settings [25, 26]. This is very relevant, as the underuse of evidence-based HF medication is associated with a higher use of healthcare resources, particularly first and recurrent hospitalizations [27].

During the period 2015–2019, patient cumulative cardiovascular disease hospital mean cost reached 11,649 Euros. Importantly, cardiorenal hospitalizations were the most important contributor for the total cost, particularly HF hospitalizations. Overall, HF hospitalizations represent 1–2% of total admissions [7, 28] and HF is the most common diagnosis in elderly hospitalized patients [29]. During the first year after diagnosis of HF, approximately half of the patients may be expected to be hospitalized at least once. In addition, readmission rates are high [7, 30–32]. Importantly, it has been reported that in Spain, rates of first hospitalization due to HF continue to increase, with high mortality [32]. A recent systematic review analyzed 16 cost-of-illness studies related to HF. Although there were large variations concerning cost components, the majority of them showed that hospital admission costs were the most expensive cost element. Annual costs for HF patients ranged from 868 Dollars (≈774 Euros) for South Korea to 25,532 Dollars (≈22,760 Euros) for Germany [3]. Other systematic review focused on economic HF burden also showed that hospitalization cost was found to be the main cost driver to the total health care cost and that the HF annual cost ranged from 908 Dollars (≈809 Euros) to 40,971 Dollars (≈36,522 Euros) per patient [12]. In our study, during the 2015–2019 period, HF associated costs per patient reached 15,373 Euros, in line with these studies. However, among other factors, methodological heterogeneity and specific cost items (including treatments) accounted for in the estimations indicate that cost comparisons across publications should be made with caution [33]. Certainly, all these data confirm the high cost burden of HF. Some factors such as age, renal function, blood pressure, NYHA functional class, diabetes, body mass index, or medication/diet nonadherence have been associated with a higher morbidity and mortality. Therefore, since HF hospitalization is the main driver for HF costs, the early identification of these patients is mandatory, as these patients require a more careful follow-up and a greater intensification of treatment, in order to reduce HF burden [34, 35].

As the most important contributor for HF cost is HF hospitalizations, the use of those drugs that have demonstrated to be beneficial in this clinical context may be very helpful in reducing total HF cost. Thus, in 2014 the PARADIGM-HF trial showed that compared with enalapril, sacubitril/valsartan significantly reduced the risk of HF hospitalization by 21% and this might have had a positive impact [9]. In our study, from 2015 to 2019, in general there was a progressive reduction of cardiovascular disease hospital cost per year, as the proportion of hospitalized patients decreased. Interestingly, there was only a slight increase in HF medication cost per year which is a small contributor for total HF cost. This is in line with previous studies that have shown a decline in standardized HF hospitalization rates in Europe and United States [36, 37]. However, absolute numbers of HF hospital admissions are expected to increase by about 50% in the following years due to the ageing of the population [7]. As a result, new drugs are needed to improve these numbers. In 2019, the DAPA-HF trial showed that in addition to recommended therapy, dapagliflozin significantly reduced the risk of a first worsening HF event by 30% [10]. Therefore, it can be hypothesized the addition of dapagliflozin to standard HF therapy may contribute to reduce HF costs.

Other contributors to total HF cost included primary care visits, specialized visits, and diagnostic tests. It has been reported that a better integrated hospital primary care HF program is associated with a significant reduction of readmission for HF and mortality [38]. In addition, moving to case management at home rather than outpatient cardiology clinic follow-up may also reduce healthcare costs [39]. Therefore, transition to an integrated management of HF patients is necessary to reduce HF burden.

A recent meta-analysis estimated the one, two, five and 10-year survival to be 87, 73, 57 and 35%, respectively, among HF patients [40]. HF hospitalization is an independent predictor for increased HF mortality [7, 30, 31]. In our study the proportion of patients who died decreased from 9.4% in 2015 to 4.9% in 2019, in line with the decrease in hospitalization rates. Although there is much room for progress, it is likely that the improvement in HF management during these years may have had a positive impact.

A specific analysis was performed in the DAPA-HF like population. In the DAPA-HF trial, the addition of dapagliflozin resulted in a significant reduction of HF hospitalizations, death from cardiovascular causes, and death from any cause, regardless the presence of diabetes [10]. In our study, in the DAPA-HF like population, all patients had reduced left ventricular ejection fraction HF and the majority of patients were on NYHA functional class II or III. Compared with the overall HF population, patients were taking more renin angiotensin system inhibitors and beta blockers. Although in these patients there was a decrease of patients hospital mean cost for year, these were higher than in the overall HF population. Thus, cumulative cardiovascular disease hospital cost reached 13,775 Euros (vs 11,649 Euros in the overall HF population). The great burden for this cost was due to cardiorenal hospitalizations (87.7% of the total hospital cost), particularly HF (65.9% of the total hospital cost). Therefore, to reduce HF cost burden in the DAPA-HF like population is of utmost importance to improve the HF management. As the great majority of these patients were taking renin angiotensin system inhibitors and beta blockers, the prescription of newer HF drugs, such as dapagliflozin, could be of particular benefit in the reduction of HF costs [10]. In fact, a recent study has shown that dapagliflozin may be a cost-effective treatment for HF patients in not only in United Kingdom and Germany, but also Spain [41].

This study has some limitations that should be commented. This was an observational cohort study that used secondary data from electronic health records. In addition, there were certain factors, such as some clinical characteristics that could not be controlled. As a result, variations in healthcare costs can not only be related with modifications in the prescription of HF drugs. Therefore, only indirect causality can be provided. However, the high number of patients included, as well as the robustness of the data may allow to determine the value of the study. On the other hand, although data came from seven Spanish regions, previous studies have shown that these data are representative of the Spanish population [19].

## Conclusion

During the 2015–2019 period, costs of patients with HF in Spain were high, being cardiovascular hospitalizations the most important determinant, particularly HF hospitalizations. Medication costs were responsible for only a small proportion of total HF costs. Costs and healthcare resources use were even higher in the DAPA-HF like population. Improving HF management, particularly through the use of those drugs that reduce HF hospitalization may be helpful to reduce HF burden.

## Supplementary information

**Additional file 1: Table S1.** Definition of variables.

**Additional file 2: Table S2.** Description of costs / units (year 2019).

**Additional file 3: Table S3.** Patient cumulative hospital mean cost*.

**Additional file 4: Table S4.** Baseline clinical characteristics of the DAPA-HF population according to the presence of type 2 diabetes.

**Additional file 5: Table S5.** DAPA-HF patients hospital mean cost for year and cumulative cost in 2019*.

## Data Availability

This was a secondary data study using BIG PAC® database. Public access to the database is open.

## Abbreviations

HF: Heart failure
T2D: Type 2 diabetes
