# Supplementary material for: Costs and healthcare utilisation of patients with heart failure in Spain
Source: BMC Health Serv Res. 2020 Oct 20;20:964. doi: 10.1186/s12913-020-05828-9 (PMC7576860; doi:10.1186/s12913-020-05828-9)
Supplement: Supplementary file 1 — Additional file 1: Table S1. Definition of variables. [file 12913_2020_5828_MOESM1_ESM.docx]

**Supplementary table 1**. **Definition of variables.**

| **Variable** | **Definition (codes)** |
| --- | --- |
| Myocardial infarction | I21, I22 |
| Stroke | I60-I63 |
| Heart failure | I50, I11.0, I13.0, I13.2 |
| CKD | N17-N19, I12.0-I2.9, I13.1, I13.2, N08.3, E10.2, E11.2, E12.2, E13.2, E14.2, Z49, Z99.2 + procedure codes |
| PAD | I70.2, I73.9, I74.2-9 |
| CVD related medications | B01AC06, C10AA, B01AA03, B01AC04, B01AC22, B01AC24, B01AC07, B01AC09, B01AC11, B01AC13, B01AC16, B01AC17, B01AC21 |
| HF related medications | C09A, C09B, C09C, C09D, C03C, C03DA, C07 |
| Diabetes related medications | All A10 medications |

CKD: chronic kidney disease; PAD: peripheral artery disease; CVD: cardiovascular disease; HF: heart failure.
