# Supplementary material for: Costs and healthcare utilisation of patients with heart failure in Spain
Source: BMC Health Serv Res. 2020 Oct 20;20:964. doi: 10.1186/s12913-020-05828-9 (PMC7576860; doi:10.1186/s12913-020-05828-9)
Supplement: Supplementary file 2 — Additional file 2: Table S2. Description of costs / units (year 2019). [file 12913_2020_5828_MOESM2_ESM.docx]

**Supplementary table 2. Description of costs / units (year 2019)**

| Healthcare and non-healthcare resources | Unit costs (€) |
| --- | --- |
| Medical visits |  |
| Medical visits and primary care | 24,20 € |
| Emergency room visit | 118,50 € |
| Hospitalization (per day) | 480,90 € |
| Specialist medical visit | 94,50 € |
| Supplementary tests |  |
| Lab tests | 32,30 € |
| Conventional radiology | 28,50 € |
| Diagnostic test/therapeutic | 37,10 € |
| Computerized axial tomography | 96,00 € |
| Magnetic nuclear resonance | 177,00 € |
| Pharmaceutical prescription | RP+VAT |
| Work productivity - Indirect costs |  |
| Cost per day not worked | 101,21 € |
| RP: retail price. |  |
