# Supplementary material for: Costs and healthcare utilisation of patients with heart failure in Spain
Source: BMC Health Serv Res. 2020 Oct 20;20:964. doi: 10.1186/s12913-020-05828-9 (PMC7576860; doi:10.1186/s12913-020-05828-9)
Supplement: Supplementary file 3 — Additional file 3: Table S3. Patient cumulative hospital mean cost*. [file 12913_2020_5828_MOESM3_ESM.docx]

**Supplementary table 3. Patient cumulative hospital mean cost*.**

|  | **2015** | **2016** | **2017** | **2018** | **2019** |
| --- | --- | --- | --- | --- | --- |
| **Total hospital cost** | | | | | |
| CVD cost | 2,834 | 5,250 | 7,518 | 9,503 | 11,649 |
| Cardiorenal cost | 2,536 | 4,690 | 6,691 | 8,450 | 10,346 |
| HF cost | 1,967 | 3,623 | 5,099 | 6,396 | 7,842 |
| CKD cost | 569 | 1,067 | 1,592 | 2,055 | 2,504 |
| MI cost | 98 | 184 | 263 | 333 | 416 |
| Stroke cost | 138 | 264 | 396 | 503 | 616 |
| PAD cost | 63 | 112 | 169 | 217 | 271 |
| **Medication cost** | | | | | |
| Total medication cost | 230 | 446 | 673 | 877 | 1,083 |
| Diabetes medication cost | 101 | 198 | 316 | 401 | 486 |
| HF medication cost | 86 | 165 | 241 | 327 | 417 |
| CVD medication cost | 44 | 84 | 117 | 150 | 181 |

*In Euros.

CVD: cardiovascular disease; HF: heart failure; CKD: chronic kidney disease; cardiorenal: HF and/or CKD; MI: myocardial infarction; PAD: peripheral artery disease.
