# Supplementary material for: Costs and healthcare utilisation of patients with heart failure in Spain
Source: BMC Health Serv Res. 2020 Oct 20;20:964. doi: 10.1186/s12913-020-05828-9 (PMC7576860; doi:10.1186/s12913-020-05828-9)
Supplement: Supplementary file 4 — Additional file 4: Table S4. Baseline clinical characteristics of the DAPA-HF population according to the presence of type 2 diabetes. [file 12913_2020_5828_MOESM4_ESM.docx]

**Supplementary table 4. Baseline clinical characteristics of the DAPA-HF population according to the presence of type 2 diabetes**.

|  | **No T2D (n=1,864; 58.7%)** | **T2D (n=1,314; 41.3%)** | **Total HF (n=3,178; 100%)** | **P*** |
| --- | --- | --- | --- | --- |
| **Biodemographic data** | | | | |
| Age, years  ≥85 years, n (%) | 76.9±12.6  990 (53.1) | 76.8±10.1  595 (45.3) | 76.9±11.7  (1,585; 49.9%) | 0.833  <0.001 |
| Gender, male, n (%) | 971 (52.1) | 661 (50.3) | 1,632 (51.4) | 0.321 |
| Systolic blood pressure (mmHg) | 129.1±22.0 | 131.5±22.5 | 130.1±22.2 | 0.005 |
| **Heart failure data** | | | | |
| NYHA functional class, n (%)  I  II  III  IV | NA  997 (53.5)  799 (42.9)  68 (3.79) | NA  657 (50.0)  567 (43.2)  90 (6.9) | NA  1,654 (52.1)  1,366 (43.0)  158 (5.0) | <0.001 |
| Left ventricular ejection fraction, %  ≤40% | 36.4±7.4  1,864 (100) | 31.7±7.9  1,314 (100) | 34.5±7.9  3,178 (100) | <0.001  - |
| **Laboratory data** | | | | |
| eGFR, ml/min/1.73 m^2^ | 75.5±20.5 | 72.2±20.7 | 74.1±20.7 | <0.001 |
| HbA1c, % | 5.2 (0.9) | 7.3 (0.8) | 6.2 (1.4) | <0.001 |
| **Comorbidities** | | | | |
| **Cardiovascular disease, n (%)** | | | | |
| Myocardial infarction | 287 (15.4) | 295 (22.5) | 578 (18.2) | <0.001 |
| CABG | 27 (1.5) | 31 (2.4) | 58 (1.8) | 0.059 |
| PCI with stent | 1 (0.1) | 52 (4.0) | 53 (1.7) | <0.001 |
| Unstable angina | 108 (5.8) | 144 (11.0) | 252 (7.9) | <0.001 |
| Angina pectoris | 142 (7.6) | 111 (8.5) | 253 (8.0) | 0.395 |
| Stroke | 171 (9.2) | 133 (10.1) | 304 (9.6) | 0.371 |
| Ischemic stroke | 136 (7,3) | 94 (7,2) | 230 (7,2) | 0,879 |
| Hemorrhagic stroke | 24 (1,3) | 9 (0,7) | 33 (1,0) | 0,099 |
| Transitory ischemic attack | 40 (2,2) | 41 (3,1) | 81 (2,6) | 0,086 |
| Atrial Fibrillation | 171 (9,2) | 133 (10,1) | 304 (9,6) | 0.371 |
| Peripheral artery disease | 141 (7,6) | 94 (7,2) | 235 (7,4) | 0.663 |
| Chronic kidney disease | 513 (27.5) | 586 (44.6) | 1,099 (34.6) | <0.001 |
| Microvascular complications | 0 | 470 (35.8) | 470 (14.8) | <0.001 |
| Diabetic mono-/polyneuropathy | 0 | 102 (7.8) | 102 (3.2) | <0.001 |
| Diabetic eye complications | 0 | 424 (32.3) | 424 (13.3) | <0.001 |
| Diabetic foot/peripheral angiopathy | 0 | 67 (5.1) | 67 (2.1) | <0.001 |
| Diabetic kidney disease | 0 | 132 (10.1) | 132 (4.2) | <0.001 |
| Diabetes with complications | 0 | 956 (72.8) | 956 (30.1) | <0.001 |
| Severe hypoglycemia | 0 | 128 (9.7) | 128 (4.0) | <0.001 |
| **Other comorbidities, n (%)** | | | | |
| Cancer | 247 (13.3) | 202 (15.4) | 449 (14.1) | 0.091 |
| Chronic obstructive pulmonary disease | 307 (16.5) | 237 (18.0) | 544 (17.1) | 0.248 |
| Lower limb amputations | 8 (0.4) | 35 (2.7) | 43 (1.4) | <0.001 |
| Major organ specific bleeding | 20 (1.1) | 40 (3.0) | 60 (1.9) | <0.001 |
| Bariatric surgery | 1 (0.1) | 1 (0.1) | 2 (0.1) | 0.804 |
| **Therapies** | | | | |
| **Heart failure medication, n (%)** | 1,864 (100) | 1,314 (100) | 3,178 (100) | - |
| Renin angiotensin system inhibitors | 1,360 (73.0) | 1,088 (82.8) | 2,448 (77.0) | <0.001 |
| Angiotensin-converting enzyme inhibitors | 731 (39.2) | 450 (34.3) | 1,181 (37.2) | 0.004 |
| Angiotensin receptor blockers | 629 (33.7) | 638 (48.6) | 1,267 (39.9) | <0.001 |
| Beta blockers | 1,864 (100) | 1,314 (100) | 3,178 (100) | - |
| Loop-diuretics | 1,159 (62.2) | 1,020 (77.6) | 2,179 (68.6) | <0.001 |
| Aldosterone antagonists | 377 (20.2) | 290 (22.1) | 667 (21.0) | 0.002 |
| Sacubitril/valsartan | 142 (7.6) | 136 (10.4) | 278 (8.8) | 0.007 |
| Digoxin | 0 | 22 (1.7) | 22 (0.7) | <0.001 |
| **Other cardiovascular medications, n (%)** | | | | |
| Low dose aspirin | 631 (33.9) | 496 (37.8) | 1,127 (35.5) | 0.024 |
| Receptor P2Y12 antagonists | 129 (6.9) | 111 (8.5) | 240 (7.6) | 0.109 |
| Statins | 837 (44.9) | 683 (52.0) | 1,520 (47.8) | 0.000 |
| Antihypertensives | 368 (19.7) | 293 (22.3) | 661 (20.8) | 0.080 |
| Dihydropyridines CCB | 283 (15.2) | 237 (18.0) | 520 (16.4) | 0.032 |
| Low ceiling diuretics (thiazides) | 48 (2.6) | 26 (2.0) | 74 (2.3) | 0.272 |
| Non-hydropyridines CCB | 55 (3.0) | 39 (3.0) | 94 (3.0) | 0.977 |
| Nitrates | 253 (13.6) | 223 (17.0) | 476 (15.0) | 0.008 |
| Warfarin | 525 (28.2) | 406 (30.9) | 931 (29.3) | 0.096 |
| **Diabetes medication, n (%)** | 0 | 1,228 (93.5) | 1,228 (38.6) | <0.001 |
| Metformin | 0 | 871 (66.3) | 871 (27.4) | <0.001 |
| Sulfonylurea | 0 | 521 (39.7) | 521 (16.4) | <0.001 |
| DPP4 inhibitors | 0 | 328 (25.0) | 328 (10.3) | <0.001 |
| SGLT-2 inhibitors | NA | NA | NA |  |
| GLP-1 receptor agonists | 0 | 11 (0.8) | 11 (0.4) | <0.001 |
| Metiglinides | 0 | 77 (5.9) | 77 (2.4) | <0.001 |
| Glitazones | 0 | 18 (1.4) | 18 (0.6) | <0.001 |
| Acarbose | 0 | 19 (1.5) | 19 (0.6) | <0.001 |
| Insulin | 0 | 344 (26.2) | 344 (10.8) | <0.001 |

T2D: type 2 diabetes; HF: heart failure; eGFR: estimated glomerular filtration rate; CABG: coronary artery bypass graft; PCI: percutaneous coronary intervention; CCB: calcium channel blockers; DPP4: dipeptidyl peptidase 4; SGLT-2: sodium-glucose Cotransporter-2; GLP-1: glucagon-like peptide-1.

*p values comparing no T2D vs T2D
