# Supplementary material for: Costs and healthcare utilisation of patients with heart failure in Spain
Source: BMC Health Serv Res. 2020 Oct 20;20:964. doi: 10.1186/s12913-020-05828-9 (PMC7576860; doi:10.1186/s12913-020-05828-9)
Supplement: Supplementary file 5 — Additional file 5: Table S5. DAPA-HF patients hospital mean cost for year and cumulative cost in 2019*. [file 12913_2020_5828_MOESM5_ESM.docx]

**Supplementary table 5. DAPA-HF patients hospital mean cost for year and cumulative cost in 2019*.**

|  | **2015** | | **2016** | | **2017** | | **2018** | | **2019** | | **Cumulative cost in 2019** |
| --- | --- | --- | --- | --- | --- | --- | --- | --- | --- | --- | --- |
|  | mean | SD | mean | SD | mean | SD | mean | SD | mean | SD |  |
| **Total hospital cost** | | | | | | | | | | |  |
| CVD cost | 3,269.6 | 5,590.3 | 2,835.5 | 6,152.8 | 2,754.5 | 5,480.7 | 2,375.9 | 6,074.9 | 2,539.5 | 5,653.6 | 13,775 |
| Cardiorenal cost | 2,913.2 | 5,134.6 | 2,487.3 | 5,049.6 | 2,397.8 | 4,764.8 | 2,050.3 | 4,749.8 | 2,225.3 | 4,880.8 | 12,074 |
| HF cost | 2,242.1 | 4,295.8 | 1,865.5 | 4,063.4 | 1,793.3 | 3,877.5 | 1,488.2 | 3,793.4 | 1,694.2 | 3,889.1 | 9,083 |
| CKD cost | 671.1 | 2,344.0 | 621.8 | 2,378.1 | 604.5 | 2,205.5 | 562.0 | 2,157.3 | 531.1 | 2,214.9 | 2,991 |
| MI cost | 156.0 | 1,011.7 | 150.0 | 1,174.8 | 120.9 | 1,029.3 | 127.4 | 1,169.4 | 123.2 | 986.7 | 678 |
| Stroke cost | 123.8 | 919.8 | 137.9 | 1,034.7 | 165.5 | 1,163.9 | 140.9 | 1,233.6 | 132.1 | 986.9 | 700 |
| PAD cost | 76.6 | 908.9 | 60.4 | 822.4 | 70.2 | 756.4 | 57.4 | 797.9 | 58.9 | 613.3 | 324 |
| **Medication cost** | | | | | | | | | | |  |
| Total medication cost | 297.8 | 472.0 | 277.6 | 479.4 | 255.0 | 505.6 | 267.4 | 564.4 | 180.8 | 500.6 | 1,279 |
| Diabetes medication cost | 128.5 | 364.3 | 122.9 | 373.5 | 105.5 | 318.4 | 108.5 | 339.5 | 74.6 | 301.2 | 540 |
| HF medication cost | 112.9 | 155.4 | 104.0 | 156.6 | 106.3 | 275.2 | 116.6 | 335.7 | 74.7 | 264.2 | 514 |
| CVD medication cost | 56.4 | 113.1 | 50.6 | 103.6 | 42.2 | 93.2 | 42.1 | 99.9 | 29.0 | 87.3 | 220 |

*In Euros.

CVD: cardiovascular disease; HF: heart failure; CKD: chronic kidney disease; cardiorenal: HF and/or CKD; MI: myocardial infarction; PAD: peripheral artery disease.
